# The challenges of national health data ecosystems in feeding the European health data space: the Italian example

**DOI:** 10.3389/fmed.2025.1644719

**Published:** 2025-12-08

**Authors:** Ylenia Murgia, Roberta Gazzarata, Mario Ciampi, Mario Sicuranza, Franco Cirillo, Christian Esposito, Norbert Maggi, Gabriella Balestra, Lucia Sacchi, Mauro Giacomini

**Affiliations:** 1 Department of Informatics, Bioengineering, Robotics and System Engineering (DIBRIS), University of Genoa, Genoa, Italy; 2 Healthropy, Savona, Italy; 3 HL7 Europe, Brussels, Belgium; 4 HL7 Italy, Rome, Italy; 5 Institute for High Performance Computing and Networking, National Research Council of Italy (ICAR-CNR), Naples, Italy; 6 Department of Computer Science, University of Salerno, Fisciano, Italy; 7 PoliToBIOMedLab, Department of Electronics and Telecommunications, Politecnico di Torino, Turin, Italy; 8 Italian Scientific Society of Biomedical Informatics (SIBIM), Pavia, Italy; 9 Department of Industrial and Information Engineering, University of Pavia, Pavia, Italy; 10 OHDSI Italian National Node, Italy

**Keywords:** European Health Data Space, health information infrastructure, DHEAL-COM, interoperability, Electronic Health Record, HL7 Fast Healthcare Interoperability Resources, Observational Medical Outcomes Partnership, General Data Protection Regulation

## Abstract

The European Health Data Space (EHDS) is an European Union (EU) initiative, aimed at helping facilitate the secure and standardized sharing of health data to improve continuity of care, research and innovation. However, the successful implementation of such an ecosystem requires the active participation and cooperation of EU Member States (MS), each of which needs to adapt its local health data infrastructure to meet the requirements at the European level. The specific characteristics of the various European countries, such as size, number of citizens, and internal organization greatly influence the ease with which a country can integrate its health data structure into this supra-national system. States with higher levels of local autonomy are experiencing significant challenges in this process. For instance, the Italian National Healthcare System (NHS) is highly decentralized, with significant variability among regions and localities. This fragmentation raises problems for health data sharing and the integration of the Italian health ecosystem into the broader EHDS framework. This paper explores Italy’s organizational and regulatory challenges, the technical barriers to interoperability, and the implications of data sharing in a decentralized environment. Moreover, by examining key Italian projects, such as Digital Health Solutions in Community Medicine (DHEAL-COM), and the tools developed by Standards Developing Organizations (SDOs) and affiliated bodies, such as Health Level 7 (HL7) Europe and Italy, the paper identifies successful initiatives and proposes strategies to overcome existing obstacles, also including security and privacy aspects of healthcare data.

## 1 Introduction

### 1.1 European context: the European Health Data Space

In the last few decades, the rise of new and advanced technologies widely fostered the design, development, and use of digital tools in many sectors, including the healthcare environment. Using technologies such as Artificial Intelligence (AI), Machine Learning (ML), cloud and telemedicine platforms is commonplace, and, nowadays, it is well known how data generated in healthcare settings and their secondary use, which involves their use to achieve different goal than the one for which they are initially collected (1), play a key role in producing new knowledge. In addition, the COVID-19 pandemic added value to the need to be able to easily retrieve health data in a timely manner to anticipate the onset of health threats or to find a cure quickly.

Against this backdrop of disease prevention and extensive clinical data production and reuse, the need for a new Regulation, named the European Health Data Space (EHDS), has arisen, both to improve the use of natural persons’ own health data and to encourage secondary use and sharing among European Union (EU) Member States (MS) for the purpose of improving the wellbeing of society, thereby facilitating the work of third parties such as health professionals, researchers, and policymakers.

Table 1 highlights the most important dates that marked the path of the creation of the EHDS. At the time of writing this article, the two most recent dates mark the turning point in the world of health data: on March 5, 2025, the EHDS Regulation was officially published in the Official Journal of the European Union (2), and entered into force on March 26, 2025, starting the gradual implementation process that will reach full completion in March 2034.

**TABLE 1:** Key dates in the development and adoption of the European Health Data Space (EHDS) Regulation.

Date	Event	References
May 3, 2022	European Commission proposal presentation for the EHDS. Official start of the legislative ITER.	(59)
December 6, 2023	European Council agreement on the EHDS proposal. Beginning of negotiations with the European Parliament.	(60)
March 15, 2024	Provisional political agreement between European Council and European Parliament on the final text of the EHDS Regulation	(61)
April 24, 2024	Official adoption of the EHDS Regulation by the European Parliament	(62)
March 5, 2024	Official publication of the EHDS Regulation in the Official Journal of the European Union.	(2)
March 26, 2024	Entry into force of the EHDS Regulation	–

The table outlines the main legislative steps taken from the initial proposal to the official entry into force of the EHDS Regulation, highlighting the timeline of political agreements and formal decisions at European level.

Chapter II and Chapter IV of the Regulation deal with the topics of primary use and secondary use of data, respectively. Regarding primary use, Article 3 describes the “Right of natural persons to access their personal electronic health data”. Specifically, natural persons have the right to access at least the priority categories of their personal electronic health data mentioned in Article 14, i.e.:

- Patient summaries;
- Electronic prescriptions;
- Electronic dispensations;
- Medical imaging studies and related imaging reports;
- Medical test results, including laboratory and other diagnostic results and related reports; and
- Discharge reports.

In a further step, with the creation of the MyHealth@EU platform, the exchange of personal electronic health data for primary use among EU MS will be made possible through the adoption of the European Electronic Health Record exchange Format (EEHRxF).

As for the reuse of health data, section 4 states that EU MS will need to be able to connect to the cross-border infrastructure for secondary use, called HealthData@EU. This platform will support several research projects by making available health data from the various EU MS.

To summarize, the EHDS Regulation aims to harmonize health data to provide individuals with better use regardless of which EU country they are in and to improve and facilitate secondary use. However, each EU MS will have to decide how to achieve compliance with the Regulation, considering its own organization of the healthcare system and other existing regulatory frameworks, especially regarding protection and privacy.

### 1.2 Overview of the organization of health management in the European countries and their future EHDS integration

The diverse political, social, and economic traditions of each EU country are reflected in the organization of their healthcare systems (3). Management models vary between centralized and decentralized systems, with direct implications for efficiency, service quality, and technology integration. Centralization and decentralization are processes through which powers, responsibilities or resources are transferred or concentrated toward or away from a central authority, respectively (4). Countries with centralized healthcare systems, such as France and Estonia, promote uniformity in services and resource distribution. In contrast, countries with more decentralized systems, like Germany, Norway, Spain, and Austria, delegate significant management to local authorities, which can lead to more personalized care and better citizen wellbeing (5). However, this can also create inequalities in service quality, accentuating geographic disparities (6).

A common challenge across Europe is the General Data Protection Regulation (GDPR) (7), which can be interpreted and applied in different ways, leading to varying levels of implementation. An example of this is Ireland, where a strict and conservative approach to GDPR and healthcare research legislation limits patient data access for both patients and research purposes (8). Similarly, while Sweden has had advanced technical solutions for healthcare data management for years, it faces restrictive legal frameworks. As a result, the country will be one of the last EU members to join the EHDS data management and sharing platforms (9). Luxembourg and France have made significant strides toward EHDS adoption: Luxembourg by establishing the Luxembourg National Data Service (LNDS) in 2022 (10) and France by launching the Health Data Hub in 2019, centralizing data access for researchers and public entities (11).

EU healthcare systems are evolving at different speeds toward digitalization and EHDS integration, with progress shaped by governance models, legal interpretations of GDPR, and varying levels of digital maturity (12), ultimately affecting both primary and secondary health data use.

### 1.3 Paper objective and structure

The aim of this paper is to analyze the main challenges to be faced, barriers to be overcome, and strategies adopted by the Italian National Health System (NHS) to align with the regulations provided by the EHDS. To achieve this, a comprehensive overview of the organizational and technical context of the Italian NHS is provided, starting with the structure and technologies currently in use, and then examining the constraints and difficulties that hinder compliance with the EU directives. Next, strategies implemented to overcome these barriers are explored, including the adoption of international standards and participation in national projects. Moreover, the important issues of privacy and security are addressed, which is essential to ensure the protection of sensitive data in the digitalization and interoperability process. Finally, a discussion of the topics covered is provided.

## 2 The organization of the Italian health care system

### 2.1 Layered organizational structure: the evolution of the Italian NHS through reforms

The current high level of decentralization in the Italian NHS and its layered structure are the result of multiple reforms. Since its beginning, the Italian Republic has recognized the right to health in its fundamental law (Article 32 of the Constitution). This principle was incorporated into Law No. 833 of 23 December 1978, which established the NHS, known as *Sistema Sanitario Nazionale* (SSN) in Italian, based on three key principles: universality, equality, and equity (13). In addition to extending universal access to healthcare services for all citizens, the law established a new administrative model based on three distinct levels (14). The central government, represented by the Ministry of Health (MoH), was responsible for national planning and financing, managed through mandatory contributions and taxes. The 20 regions of Italy, on the other hand, were responsible for local planning and organizing healthcare services within their territories. Finally, about 650 Local Health Units (LHUs) (15), in Italian *Unità Locali Sanitarie* (USL), were responsible for directly providing services, managing their own structures or contracting with private providers.

This model showed two major shortcomings: excessive politicization, because management responsibilities at the LHU level were concentrated in the hands of political bodies, often lacking technical expertise; and the lack of rational financial incentives, which led to continuous conflict between the central government and the regions (14). The response to these limitations was the beginning of the decentralization process (16). Through a series of reforms (Legislative decree No. 502/1992, Legislative decree No. 517/1993, and Legislative decree No. 229/1999) from the 1990s, the authority of the regions was strengthened, and the concept of corporate management was introduced. The LHUs were reduced in number and replaced by Local Health Authorities (LHAs), in Italian *Aziende Sanitare Locali* (ASL), which were divided into health districts, while larger and specialized hospitals became semi-independent hospital trusts, in Italian *Aziende Ospedaliere* (AO). Both LHAs and AO were made directly accountable to the regions, which gained greater autonomy in managing healthcare services.

The reform of part of the Italian Constitution (Law N. 3/2001) marked the culmination of this decentralization process, redefining the competencies between the central government, which retained some directive functions such as defining the essential assistance levels, known as *Livelli Essenziali di Assistenza* (LEA) in Italian, and the regions, which became responsible for financing, regulating, organizing, and delivering healthcare services (16). These changes shaped the current structure of the Italian NHS, where regions are now accountable for the political, administrative, and financial aspects of providing healthcare to their local citizens (17). As a result of this high decentralization, Italy now has 20 Regional Healthcare Systems (RHSs), each with its own governance model and management strategies (18–20). Despite this autonomy, the allocation and supervision of financial resources remain closely linked to the Ministry of Economy and Finance (MEF), which plays a central role in coordinating the national health budget (21). Each year, the MEF distributes health funds to regions based on predefined criteria, often related to demographics, historical spending, and health needs (22). This is particularly relevant also in the context of Health Information Infrastructure (HII), which aims at ensuring that complete Electronic Health Records (EHRs) are accessible whenever and wherever they are needed across the entire population (23). In Italy, the equivalent of HII is the *Fascicolo Sanitario Elettronico* (FSE): although implemented at regional level, it is part of a nationally coordinated effort in which the MEF is directly involved in ensuring that digital infrastructures align with fiscal accountability frameworks and interoperable standards. For the purposes of this paper, we will refer to the Italian HII using the acronym FSE as it is explicitly used in many Italian projects.

More recently, in April 2021, due to the COVID-19 pandemic, which highlighted the need for greater digitalization of the Italian NHS (16), the National Recovery and Resilience Plan (NRRP) (24) was established to promote the economic and social recovery of Italy. This topic will be explained in more detail in Section 3.2.

### 2.2 Regulations in force on the Italian national FSE

Italy has implemented a comprehensive national framework for the National FSE, to support the digital transformation of its healthcare system. Established under Decree-Law No. 179/2012, and detailed in the Prime Ministerial Decree (DPCM) of September 29, 2015, the FSE is defined as the digital collection of health and social-health data and documents related to clinical events concerning an individual, available nationwide to ensure continuity of care and improve healthcare delivery. The DPCM delineates the structure, contents, and governance model of the FSE, defining the responsibilities of key stakeholders, and establishing that it must be unique, personal, accessible by authorized subjects, and populated with standardized metadata referring to standardized documents to ensure semantic and syntactic interoperability. Technical guidelines (25) define the required standards for document encoding (e.g., HL7 Clinical Document Architecture – CDA), metadata structuring, communication protocols, based on Integrating the Healthcare Enterprise (IHE) Cross-Enterprise Document Sharing (XDS.b) profile, and secure data exchange protocols, essential to harmonize the heterogeneous systems used across the country and enable seamless integration.

With regards to the framework architecture, each Italian Region and Autonomous Province has its own FSE Information Technology (IT) system able to store in a registry metadata related to clinical documents and data referred to its patients (26, 27). Interoperability among these decentralized systems is enabled by the National Interoperability Infrastructure, known as *Infrastruttura Nazionale per l’Interoperabilità* (INI) in Italian, which serves as the central interoperability hub. The INI connects the various regional FSE IT systems managed independently by Italy’s Regions and Autonomous Provinces, enabling the nationwide exchange and accessibility of clinical information. This ensures that patient health data can be accessed across the national territory, regardless of where the data was originally generated or where care is delivered (26, 27). Operationally, the INI does not store clinical content but functions as a federated query and routing service. It determines the correct regional source for each request and securely retrieves the relevant documents. This architecture enables a federated yet integrated health information exchange model, promoting data sovereignty at the regional level while ensuring national-level accessibility and cohesion.

The evolution of the FSE was further accelerated by the adoption of two decrees (Ministerial Decree of September 7, 2023, and Ministerial Decree of December 31, 2024), aligning with the objectives of the NRRP. Known as FSE 2.0, under the technical coordination of the Department for Digital Transformation, these reforms aim to strengthen interoperability, enforce data and format standardization, and introduce the Healthcare Data Ecosystem, known as *Ecosistema Dati Sanitari* (EDS) in Italian. This national initiative seeks to foster a connected and interoperable environment, with the introduction of a new architecture component named Gateway, where health data can be seamlessly shared among healthcare professionals, administrative bodies, and, most importantly, with the citizens themselves, all while adhering to stringent security and privacy regulations. Key objectives include the standardization of data formats, the deployment of secure exchange platforms, and the establishment of clear governance frameworks. The development of this robust national ecosystem is not only intended to modernize the Italian healthcare system and to foster a shift from documents to data, but also to strategically position Italy to engage effectively with broader European initiatives in the realm of health data sharing, such as the EHDS. By building a strong and interoperable foundation at the national level through the FSE and its associated ecosystem, Italy aims to be a proactive and valuable participant in the future landscape of cross-border.

#### 2.2.1 Alignment between EHDS and Italian infrastructure

The evolution of the FSE and the establishment of the EDS are closely aligned with the objectives of the EHDS. Both frameworks emphasize interoperability, standardization of data formats, and the secure and transparent exchange of health information, ensuring that citizens can access and share their health data across borders. The Italian approach, with its federated model based on the INI and the introduction of the Gateway component in FSE 2.0, mirrors the EHDS vision of a decentralized yet interconnected ecosystem, where national systems remain sovereign but are seamlessly integrated into a European-level infrastructure. Moreover, the emphasis on semantic and syntactic interoperability in the Italian guidelines, particularly through the adoption of HL7 CDA, HL7 FHIR, and secure communication protocols, anticipates the technical requirements being defined at the EU level.

### 2.3 Constraints and barriers

While the decentralization of the NHS allows regions greater autonomy, increasing their decision-making authority to respond to the health needs of the local population (5), the other side of the coin hides obvious problems of interoperability and standardization among the various Health Information Systems (HIS), both nationwide, but also within the same region. To the best of our knowledge, to date there is no official census quantifying the heterogeneity, but the problem is palpable and real. To confirm this point, in 2022, the Italian Higher Health Council, the senior advisory body of the MoH, published the “Proposal for the outline of the Reform of Health Information Systems” (*Proposta per lo schema di Riforma dei Sistemi Informativi Sanitari*, in Italian) highlighting the shortcomings and proposing realistic solutions (28). In this Proposal, an overview of the current state of regional and national HIS is described, and it is stated that, in Italy, there are multiple HIS that have been activated at different times, with different objectives, and having different territorial coverage. These HIS were activated independently of each other, causing the clinical data to have dissimilar organization and structure. To solve this problem, the proposal recommends adopting the HL7 Fast Healthcare Interoperability Resources (FHIR) model, which allows for a standardized data structure, while also ensuring interoperability for data reuse. This proposal represents a step in the right direction, but the issue of interoperability remains highly problematic to date. As pointed out in a recent editorial in The Lancet Regional Health – Europe (29), the health data system in Italy is defined as “broken” mainly because of the absence of unified governance. Italy’s 20 regions operate with their own, often incompatible policies, infrastructures and information systems, making even it difficult to ensure seamless communication and consistent interpretation of health information. This fragmentation hinders the sharing of clinical data among health facilities and compromises the timeliness and efficiency of care. In addition, Law No. 86, approved on June 26, 2024, contains provisions for enhancing the Regions autonomy, leading to greater decentralization of healthcare responsibilities, enhancing regional decision-making power also over digitalization. This would undermine efforts for true national harmonization and for linking the Italian system with European initiatives such as the EHDS, which requires interoperability, semantic consistency, and uniformity in data access and sharing procedures.

The current decentralized model has led not only to syntactic inconsistencies but also to semantic differences, such as the persistent use of different terminologies and vocabularies across regions and even within the same region. Hypothetically, if all HIS adopted the same standard to solve the syntactic interoperability problem, semantic interoperability would remain a stumbling block to overcome. Indeed, a further problem underlined in the Proposal is the use of information coding systems that are not perfectly identical, not only at the national level, but also at the regional level. Although Italian is Italy’s official language, there are numerous dialects that vary from region to region. This social aspect, combined with the decentralization of the NHS, meant that local terminologies and coding were adopted during the implementation of the first HIS. To better understand the level of fragmentation, consider the Veneto region, located in northern Italy, as an example. As a result of a 2016 Regional Reform (30), Veneto’s Regional Healthcare System (RHS) is currently divided into 9 LHAs and 3 autonomous hospital trusts, 2 of which are university-based and one specialized in oncology. The 9 LHAs are divided into districts, which organize and coordinate primary care services. Currently, there are 26 districts, which corresponds to the previous number of LHAs in the Veneto region before the reduction with the Regional Reform (31). While, from an operational point of view, the number of LHAs decreased, the same cannot be said for the number of coding systems used, potentially resulting in still having 26 different local coding systems.

## 3 Technical aspects

### 3.1 International and national standards

The growing complexity of healthcare systems and the imperative for seamless data exchange across institutional and national boundaries have underscored the importance of standardized frameworks in health informatics. Within this landscape, several key organizations and standards play a pivotal role in enabling semantic and technical interoperability, ensuring that clinical data is shared, interpreted, and reused consistently across diverse platforms. Table 2 lists the main standards and national and international code systems used in the Italian context to support both the semantic and syntactic interoperability of clinical and administrative data.

**TABLE 2:** Overview of the principal national and international standards, organizations, and coding systems adopted within the Italian healthcare context to ensure semantic and syntactic interoperability.

Standard/coding system	Description, role, and application
HL7 (V2.x, V3, FHIR)	HL7 International (63) is one of the most influential global standard development organizations in health informatics. It is responsible for defining data exchange protocols, such as HL7 Version 2.x, Version 3, and the increasingly adopted HL7 FHIR (Fast Healthcare Interoperability Resources). These standards provide structured formats and APIs to support the integration of EHR systems, clinical workflows, and health information systems. HL7 Europe (64) is the European office of HL7 International, established in 2010 in Brussels to address European standardization requirements in digital health by supporting the 22 national HL7 Affiliates across Europe. HL7 Italy (65) serves as the national affiliate of HL7 International, promoting the adoption of HL7 standards within the Italian healthcare system. It adapts international specifications to local legal, linguistic, and organizational requirements, while fostering alignment with national strategies such as the FSE and PNRR. Building upon the foundational work of HL7 International, both HL7 Europe and HL7 Italy play critical roles in promoting and adapting these standards within their respective contexts. HL7 Europe focuses on the adoption and implementation of HL7 standards across the European Union, facilitating cross-border interoperability and supporting initiatives like the EHDS. This includes developing European-specific implementation guides and fostering collaboration among Member States. In the Italian context, HL7 Italy plays a key role in the localization and implementation of international healthcare interoperability standards. This organization is responsible for contextualizing HL7 specifications to align them with Italian legislative decrees and specific national healthcare requirements. A key function of HL7 Italy is the development of national Implementation Guides (IGs), which provide detailed technical specifications and best practices for the adoption of HL7 standards within national initiatives such as the FSE 2.0 and the EDS. This effort includes the creation of Italian IGs for representing clinical contents in digital structured documents based on the HL7 V3 CDA standard and, with increasing emphasis, for representing and exchanging clinical digital structured data using the FHIR standard. The national IGs standardized include patient summary, laboratory report, hospital discharge letter, radiology report, prescription, vaccination, and many others.
IHE	The IHE initiative (66) complements HL7 by focusing on the orchestration of clinical use cases through implementation profiles. Among these, IHE XDS (Cross-Enterprise Document Sharing) is particularly relevant in contexts such as Italy’s federated health data infrastructure. XDS defines a set of technical specifications for the registry and retrieval of clinical documents across health enterprises which belong to an affinity domain, supporting the integration of distributed EHRs. IHE XDS is widely used in national and regional architectures to facilitate document-centric interoperability, particularly for imaging, discharge summaries, and laboratory reports. It ensures that healthcare professionals can access the most relevant and up-to-date patient information regardless of where it was originally produced.
LOINC	Logical Observation Identifiers Names and Codes (LOINC) (67) is a universal standard for identifying clinical observations, laboratory results, and measurements. Developed by the Regenstrief Institute, it assigns unique codes to each type of test or observation, ensuring consistent naming and classification of clinical data. In Italy, with the support of LOINC Italy (68), LOINC is progressively being adopted for the semantic harmonization of laboratory and diagnostic datasets, enabling cross-regional comparability and integration into national registries and the FSE (69).
ICD	The International Classification of Diseases (ICD) (70), maintained by the World Health Organization (WHO), provides a comprehensive coding system for diagnoses, symptoms, and causes of death. Italy currently utilizes ICD-9-CM for clinical coding in many administrative and reporting processes, although a transition toward ICD-10 or ICD-11 is under consideration in line with international trends. ICD codes are fundamental for epidemiological surveillance, hospital discharge summaries, and public health planning.
AIC—ATC	The *Autorizzazione all’Immissione in Commercio* (AIC) code (71), issued by the Italian Medicines Agency (AIFA), uniquely identifies medicinal products authorized for the Italian market. It is essential for tracing prescriptions, dispensing, and pharmacovigilance. Complementary, the Anatomical Therapeutic Chemical Classification (ATC) classification (72), maintained by the WHO Collaborating Centre for Drug Statistics Methodology, categorizes medications based on their therapeutic use and chemical characteristics. Together, AIC and ATC support the interoperable representation of pharmaceutical data, enabling consistent recording of prescriptions within health information systems and the FSE, and facilitating integration with electronic prescribing and medication management systems.

Each entry highlights its role, scope of application, and relevance to national initiatives.

The integration of such standards described in Table 2 forms the backbone of modern health informatics infrastructures. Their harmonized use ensures data quality, interoperability, and semantic clarity, which are essential for supporting clinical workflows, public health monitoring, and health data reuse. Organizations such as HL7 International and HL7 Europe, IHE, and national entities like HL7 Italy and *Agenzia Italiana del Farmaco* (AIFA) play a vital role in sustaining and adapting these standards to meet the evolving needs of healthcare systems, both nationally and globally.

### 3.2 Italian founded programs

The COVID-19 pandemic highlighted significant limitations and challenges within the Italian NHS, including the evident need to improve the infrastructure of healthcare facilities (32). The path toward NHS transformation is made possible by the 6-year NRRP (24), within the framework of the Next Generation EU (NGEU) program, a financial tool to support recovery in EU MS. On April 27, 2021, the Italian Parliament approved the government’s proposal for the NRRP, which was later confirmed by the EU Commission in June 2021 and by the EU Council in July 2021. The NRRP, not only offers substantial financial resources, with 194.4 billion euros allocated in loans and grants (33), but also provides a strategic vision to address also the structural challenges of the Italian NHS, defining six key Missions:

- Digitalization, Innovation, Competitiveness, and Culture.
- Green Revolution and Ecological Transition.
- Infrastructure for Sustainable Mobility.
- Education and Research.
- Inclusion and Cohesion.
- Health.

In this context, the most relevant Mission is the one related to Health, to which the NRRP has allocated 15.63 billion euros. The Health Mission is divided into two main Components: M6C1, concerning “Proximity networks, structures, and telemedicine for territorial healthcare assistance,” and M6C2, concerning “Innovation, research, and digitalization of the national health service.” The interventions related to the first Component aim to strengthen the services offered in the territory, through the improvement and creation of local structures and points of reference, the expansion of home care, innovation in telemedicine, and more effective integration between social and health services. On the other hand, the Component M6C2 focuses on the renewal and modernization of existing technological and digital infrastructures, the completion and dissemination of the Italian FSE, and improving the capacity to provide and monitor the LEAs through the adoption of more efficient information systems. Additionally, part of the resources will be allocated to scientific research and technology transfer, with the goal of promoting innovation. Finally, M6C2 also includes the strengthening of skills and human capital in the NHS through targeted initiatives to enhance personnel training.

Thanks to the introduction of Decree-Law No. 59 of May 6, 2021, the Italian government activated the Complementary Nation Plan (CNP) to support and reinforce the NRRP, allocating an additional 30.6 billion euros in national resources. This decree identifies 30 interventions, divided into 24 programs fully financed by the CNP and 6 programs co-financed with the NRRP. Regarding the health sector, the allocated resources amount to 2.89 billion euros.

Started in 2023, the Digital Health Solutions in Community Medicine project, known as DHEAL-COM (34), is an example of a program exclusively funded by the CNP. The overall goal of the DHEAL-COM project is to create a national life science hub, named DHEAL-COM HUB, designed as a reference model to develop and strengthen digital technologies to support community-based healthcare. This hub is envisioned to align with the broader EDS. By aligning with the EDS, the hub can contribute to the ongoing efforts to enhance data-driven innovation in digital health. The hub aims to become a national reference facility for research and innovation in digital health technologies, focusing on customized solutions designed around the needs of primary users (e.g., target populations), secondary users (e.g., caregivers), tertiary users (e.g., professionals, policy makers and industry) and local communities, with direct involvement of health technology companies. At the end of the project, expected outcomes include: (i) an innovative reference model for community-based digital health services to guide the integration of digital tools and management of new health services; (ii) a centralized smart repository, where project documentation, health data collected during clinical trials, mobile applications for health monitoring, and Artificial Intelligence (AI) algorithms for risk stratification and predictive analysis will be stored; (iii) a web-based open platform for citizens and stakeholders to access and use the digital tools developed during the project; (iv) an open lab, equipped with advanced hardware and software to test innovative healthcare solutions in realistic settings.

To address the challenges of semantic and structural interoperability, DHEAL-COM adopts a two-level harmonization strategy. At the local nodes, health data from partner repositories are converted into FHIR-compliant resources. At the central repository, a dedicated ETL component transforms the de-identified FHIR data into the Observational Medical Outcomes Partnership (OMOP) Common Data Model (CDM) to represent and manage health data in its processed data storage layer, thus enabling future reuse across contexts. This design choice reflects DHEAL-COM’s ambition to go beyond national impact and integrate into broader health data ecosystems. The OMOP CDM, maintained and promoted by the Observational Health Data Sciences and Informatics (OHDSI) community, provides a standardized data structure and common terminologies for observational healthcare data (35). OHDSI (36) is an open science initiative involving an international network of researchers and data partners, who focus on methodological research, open-source analytics, and clinical applications to advance the generation and dissemination of reliable medical evidence from observational data (37). OMOP CDM, besides being an open community standard for representing the structure and content of observational data (38, 39), it improves interoperability by standardizing both data structure and language, and enabling the sharing of analytics methods (40). Its use across multiple partners enables studies to be consistently developed, executed, and replicated. This has led to the development of a library of analysis packages that can be used by any researcher with data in the OMOP CDM format, and that are included in the OHDSI Health Analytics Data-To-Evidence Suite (HADES) (41). In Europe, the European Health Data and Evidence Network (EHDEN) project, funded by the Innovative Medicines Initiative 2 Joint Undertaking, has promoted OHDSI principles by creating a federated network, with 21 Italian hospitals joining as data partners. Within the OHDSI collaboration, numerous studies have applied these shared datasets and methods, focusing on treatment patterns (42), clinical pathways (43), or disease trajectories (44) demonstrating the replicability of the analysis across several datasets from around the world. Thanks to some rapid collaboration calls that were published to contrast the COVID19 pandemics, several hospitals and clinical centers had the incentive to adhere to the network, and this is acknowledged by the high number of publications generated both in the OHDSI community (45) and within EHDEN (46) in this regard.

#### 3.2.1 Alignment between EHDS and DHEAL-COM

**FIGURE 1:**
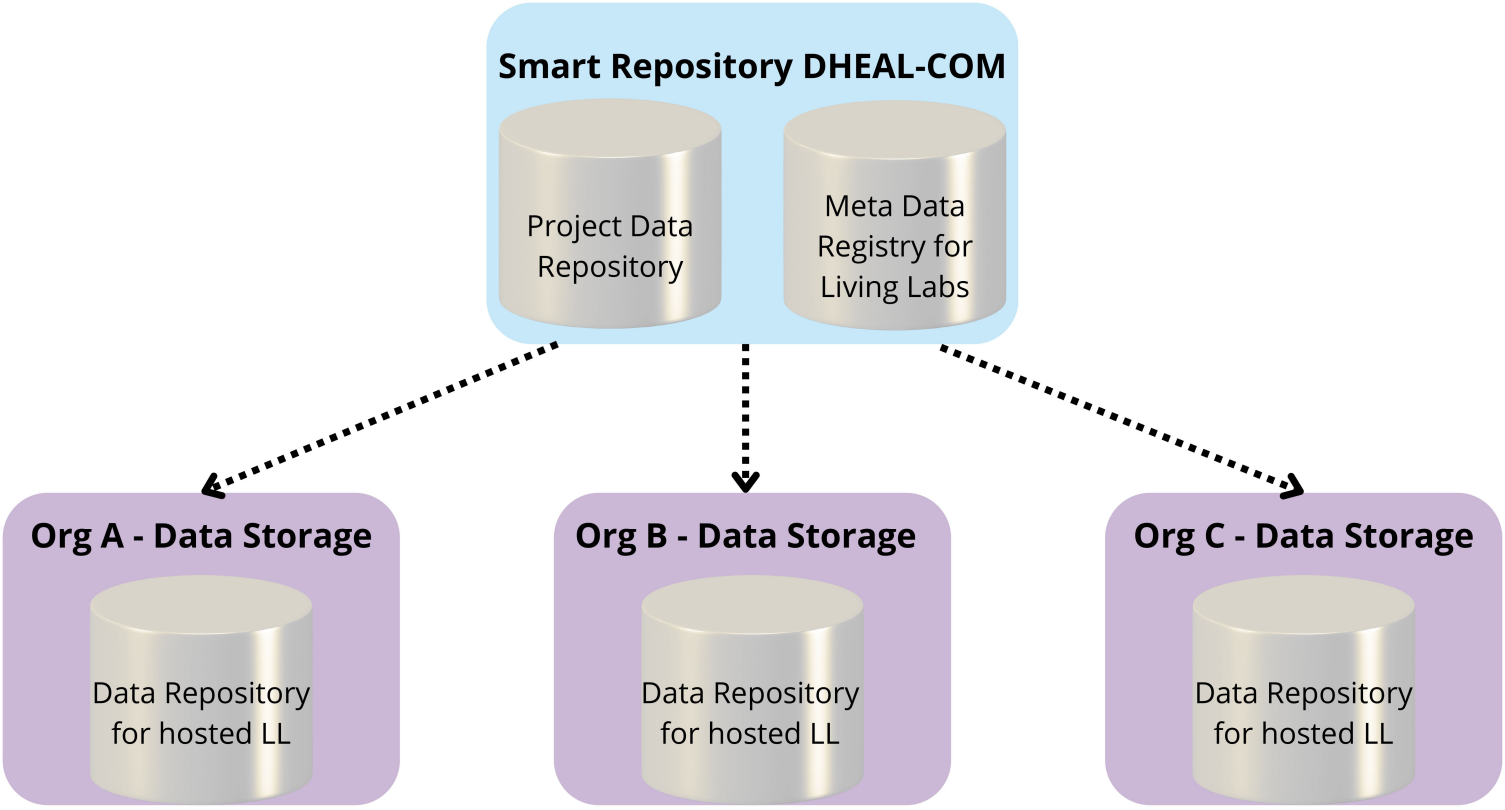
High-level architecture of the distributed DHEAL-COM smart repository. The diagram illustrates the interaction between local nodes, managed independently by each partner organization, and the centralized repository hosted by the project’s Work Package (WP) leader. Local nodes are responsible for acquiring, processing, and storing personal health data, while the central repository stores non-personal project data and associated metadata. This architecture enables federated queries while preserving data localization and compliance with privacy regulations. Note that within DHEAL-COM, metadata is stored centrally only for non-personal data and federated queries, while sensitive metadata remains local.

As described in section 1.1, the EHDS anchors two complementary infrastructures: one for the primary use of healthcare data, and the other for research-facing secondary use, where data fall under a single, interoperable rulebook and harmonized governance and security controls. DHEAL-COM is evolving in parallel with the EHDS framework, and the project has been designed with EHDS alignment in mind from the outset. A federated architecture in Figure 1 fits this model naturally because the EHDS is designed to minimize data movement, keep identifiable records inside trusted environments, and let authorized users query across multiple holders without copying raw personal data to a central repository. In an EHDS-aligned federation, a central broker operates only on metadata (capabilities, standards coverage, quality indicators, governance policies and access conditions) to decide where and how a request should be run, while execution occurs locally within each node’s secure processing environment; only non-identifying aggregates or approved, de-identified outputs leave the node. That approach mirrors HealthData@EU’s emphasis on secure access, standardized semantics and auditable controls, and it aligns with the timeline that brings obligations online progressively after entry into force. DHEAL-COM’s hub-and-spoke model can serve as a HealthData@EU-ready federation: semantics can be harmonized to common models and vocabularies, governance can track the EHDS’s access permits and safeguards, and cross-site analytics can proceed without the hub ever touching raw personal data.

### 3.3 Working tables and coordination initiatives

The successful implementation of the EHDS and the establishment of a truly interoperable EDS in Italy are inextricably linked to the effectiveness of standardization efforts. A crucial element to achieve meaningful standardization lies in the creation and active participation in joint collaborative platforms that bring together national and European actors. These synergistic interactions are vital for several key reasons. First, ensuring alignment and preventing divergences is crucial. Without integrated work, local initiatives risk developing in isolation, with potential incompatibilities with the overall European framework envisaged by the EHDS. Joint tables provide a forum for timely engagement, allowing national stakeholders (e.g., the MoH, regional health authorities, HL7 Italy, SDOs, research bodies and industry representatives) to understand the evolving requirements and technical specifications of the EHDS. At the same time, such strong integration can lead to European decision makers (e.g., the European Commission, HL7 Europe) gaining valuable insights into the specific challenges, priorities and infrastructures in EU MS such as Italy. This two-way communication facilitates the development of standards that are broadly applicable and realistically implementable at the national level, minimizing the need for costly and time-consuming adaptations.

Furthermore, addressing specific national complexities in a European context is crucial for countries with decentralized health systems like Italy. The Italian NHS, with its significant regional autonomy, presents unique challenges for standardization. Joint roundtables provide a platform to discuss these specific complexities and develop customized approaches that respect regional diversity while aligning with broader European goals. For example, current discussions focus on how to implement common data models and exchange formats in a way that considers the current diversity of local health information systems.

In this context, the National Contact Point for eHealth (NCPeH) (47) is a standardized digital infrastructure designed to facilitate the secure and interoperable exchange of health data across EU MS. Operated under the framework of the eHealth Digital Service Infrastructure (eHDSI), the NCPeH serves as a national gateway, ensuring that electronic health information–such as Patient Summaries and ePrescriptions–can be exchanged seamlessly between countries in compliance with European regulations and standards.

Technically, each NCPeH instance functions as a node within a decentralized network, translating national healthcare data formats and terminologies into a common, structured, and semantically interoperable format defined by the OpenNCP specifications (48). This translation ensures consistency and accuracy in cross-border healthcare communication.

The system architecture relies on a dual-gateway approach, composed of a Sending and a Receiving NCPeH, each responsible for handling outgoing and incoming health data, respectively. Secure data transfer is guaranteed through end-to-end encryption, digital certificates, and adherence to the eIDAS regulation for trusted electronic identification and authentication.

In practice, when a patient receives care in another European country, the local healthcare provider can request relevant health data from the patient’s home country via the NCPeH, ensuring timely access to critical medical information while maintaining data privacy, integrity, and legal compliance.

Fostering innovation and forward-looking standards is an ongoing need. The healthcare landscape and related technologies are constantly evolving. Collaboration between national and European actors can ensure that standardization efforts remain agile and forward-looking. Sharing insights into emerging technologies and future trends, ensuring the long-term sustainability and relevance of national and European health data ecosystems.

## 4 Educational aspect: training and acquisition of skills

HL7 Italy is a pivotal player in healthcare professional development, offering courses that equip individuals with the essential skills to work with some of the industry’s key standards. The objective is twofold: to impart technical knowledge and to cultivate a genuine community of professionals who are eager to contribute to working tables. This is facilitated by the preparation offered by the courses, leading to the development of new capabilities.

Specifically, HL7 Italy proposes Italian editions of two HL7 International courses delivered in e-learning mode (49): HL7 Fundamentals Course and FHIR Fundamentals Course. The first covers topics ranging from markup and modeling languages, such as UML and XML, to HL7 V2.x and HL7 CDA standards. The latter explores the world of FHIR in depth, covering topics such as resource types, the RESTful FHIR approach, and profiles. Each course concludes with an examination, and upon successful completion of all tests, a certification with international validity is awarded. Notably, while the courses are managed by HL7 Italy, they are designed and structured globally by HL7 International.

**FIGURE 2:**
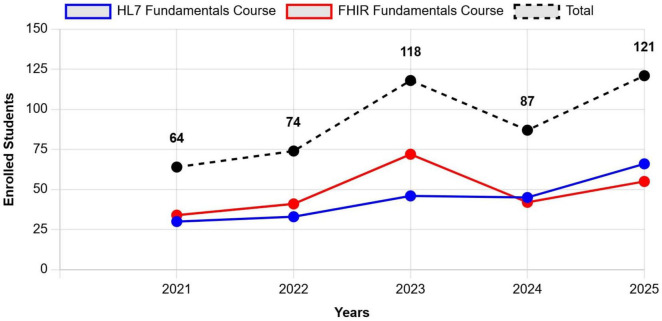
Number of participants enrolled in HL7 Italy’s courses (specifically HL7 Fundamentals Course and FHIR Fundamentals Course), over a 5-year period, from 2021 to 2025. The trend demonstrates a steady increase in participation, particularly from 2023 onwards, which aligns with the implementation phase of NRRP funding. Data for 2025 reflects actual registration, as all courses are conducted during the first semester of the year.

The trend of participation in the courses over the past 5 years has tended to grow (Figure 2), confirming a rise in interest. A significant increase was observed in 2023, primarily due to the approval of the NRRP in 2021, the subsequent release of funds in 2022, and the effective utilization of these resources starting in 2023. The increased availability of financial resources from NRRP M6C2, has facilitated greater investment in training initiatives, allowing more professionals to access specialized courses and gain expertise in standards such as FHIR, ultimately contributing to the broader digital transformation of the national health system. In addition, for both courses, HL7 Italy supports participation through scholarships, particularly for recent graduates and PhD students specialized in the issues related to the topics covered. Between 2021 and 2025, a total of 11 scholarships has been awarded: eight for the FHIR course and three for the HL7 introductory course.

From a university education perspective, the topics associated with digital healthcare are usually part of biomedical engineering or bioengineering curricula. Only a few bachelor programs have courses in biomedical informatics, while 10 out of 26 master curricula contain tracks completely devoted to it and most of the others have at least one course (these data refer to the 2024/2025 academic year). Some of these tracks are centered on medical software and medical device software and describe the design and development processes, regulations, and application environments requirements. Others focus on specific applications such as home care based on telemedicine tools or personalized medicine. Several courses are dedicated to artificial intelligence applications, mostly machine learning and deep learning. Health data are fundamental for all types of courses, both for the variety of data types often used together and the reliability that is essential for patient safety.

Although biomedical engineering is the field that typically addresses digital healthcare topics, courses can also be found in computer science, medicine and healthcare professions curricula, as well as in PhD courses and theses in bioengineering area and other disciplines.

## 5 Security aspects

### 5.1 Healthcare data in the context of GDPR

Healthcare data are universally considered uniquely sensitive and critical within the broader personal data landscape (50). There have been efforts to digitalize healthcare data and improve their interoperability among different HIS and organizations due to the increasing reliance on digital technologies in medical practice (51, 52). In this context, it is becoming equally essential to safeguard health-related information from possible misuses and violations of people’s fundamental rights.

When speaking about the impact of GDPR (7) on the proper handling of healthcare data, the starting point is Article 4(15), which identifies health data as a specific subset of personal data. This subset encompasses all aspects related to a natural person’s physical or mental health, including indirect information such as healthcare services received, which can provide insights into health status. Healthcare data is valued as highly risky and requires heightened protection, so its processing is, by default, prohibited under Article 9(1). However, Article 9(2) outlines a series of exceptions to this prohibition. These exceptions apply if a proper legal basis is correctly assumed to justify the acquisition, storage and processing of this special class of personal data. These scenarios include processing for care purposes, reasons of substantial public interest, and public health emergencies. Furthermore, according to Article 9(3) and Recital 53 of the GDPR, and Article 75 of the Italian Privacy Code, health data must be processed by a healthcare professional under confidentiality, or by others legally bound to secrecy. The Italian Data Protection Authority clarifies that in such cases, no consent is needed from the patient if the processing is necessary for medical care—regardless of whether the professional is self-employed, employed, or works in a public or private facility. This reflects the GDPR’s approach, which relies less on consent compared to prevailing Directive 95/46/EC. However, if the data is processed for purposes beyond medical care (even by healthcare professionals), a different legal basis must be used, including, when required, the patient’s explicit consent under Articles 6 and 9(2). Table 3 provides concrete examples of processing activities that do not fall under necessary care-related purposes and therefore require data subject’s explicit consent. Regardless of the specific digital application, the first requirement for healthcare providers is to ensure that the legal basis for data processing of the application is clear, documented, and appropriate to the specific use case. The other requirements are derived from the principles listed by Article 5 and are outlined in Table 4.

**TABLE 3:** Common scenarios where the processing of health-related personal data does not fall under necessary care as defined by the General Data Protection Regulation (GDPR) and the Italian Data Protection Authority (*Garante*).

Use case
Medical apps that collect personal health data for purposes other than telemedicine or any scenario in which third parties may access the data.
Health data being processed for non-essential purposes or marketing and promotional activities carried out by private legal entities in the healthcare sector.
Healthcare professionals processing data for commercial or electoral campaigns.
EHR data processed for non-care purposes (e.g., research, marketing).

These activities, such as commercial use, marketing, or third-party access, require the explicit consent of the data subject before processing can occur.

**TABLE 4:** Principles that must be followed when handling personal data under Article 5 of the GDPR.

Article	Principle	Description
5(1)(a)	Lawfulness, fairness and transparency	Patients must be informed of data processing in a comprehensible manner (via privacy notices).
5(1)(b)	Purpose limitation	Data must be collected/processed for specified, explicit, and legitimate purposes.
5(1)(c)	Data minimization	Only data necessary for treatment or care should be processed so that when the purpose is completed the data needs to be removed.
5(1)(d)	Accuracy	Medical records must be accurate and up to date, so that in case of errors an individual must be able to make corrections.
5(1)(e)	Storage limitation	Data should not be retained indefinitely but a maximum retention period needs to be identified no greater than the end of the purpose for its acquisition.
5(1)(f)	Integrity and confidentiality	Adequate technical and organizational security measures must be implemented to protect data from possible writing and/or reading from unauthorized entities.
5(2)	Accountability	Data controllers must demonstrate compliance to the legal framework through documentation, policies, and controls.

Together, they provide a framework to ensure that health data is processed transparently, securely, and for legitimate purposes.

### 5.2 The role of the Italian Data Protection Authority

The Italian legal framework for data protection is deeply influenced by the GDPR, but it also includes sometimes stricter national adaptations under the Italian Data Protection Code (Legislative Decree 196/2003, amended by Legislative Decree 101/2018). This reflects national legal traditions and sector-specific needs, with rules such as the minimum age of 14 for social media consent, criminal liability for data violations, and specific guidelines issued by the Italian Data Protection Authority (DPA), known as *Garante*.

Before the GDPR, processing sensitive data, especially in healthcare, often required prior authorization from the *Garante*. Even under the GDPR, the *Garante* maintains a proactive approach, requiring prior consultation under Article 36 for many high-risk activities and applying restrictive interpretations, particularly regarding secondary use of healthcare data without renewed consent, data reuse, or extra-European transfers. Despite Article 89, detailed Data Protection Impact Assessments (DPIAs), ethics approvals, and strong safeguards like pseudonymization are still mandatory. The *Garante* sets strict criteria for anonymization and often considers re-identification risks too high, which can delay projects, raise compliance costs, and hinder data sharing under EHDS or Horizon Europe initiatives.

An innovative application has recently gained attention: synthetic data is considered a solution to balance privacy protection and data-driven research. According to the European Data Protection Supervisor, “synthetic data is artificial data generated from original data and a model that is trained to reproduce the characteristics and structure of the original data,” and “this means that synthetic data and original data should provide very similar results when subjected to the same statistical analysis.” In healthcare, they enable data sharing for research while preserving patient confidentiality, and they support AI training without legal or ethical risks tied to personal data. Under Recital 26, truly anonymized or synthetic data fall outside its scope. However, the *Garante*, while not yet issuing specific guidance, maintains a cautious approach: projects must demonstrate that re-identification risks are insignificant, especially when using real data. Depending on the context, ethics and DPIA approvals may still be required.

### 5.3 Data security in innovative projects

The original design of the data lake for the DHEAL-COM project (34) is based on two components that form the foundation of the smart repository. The first component concerns the collection of project-related data, including documentation, dissemination materials, and source code. The second component consists of multimodal data gathered through the project’s living labs. These data have undergone a process of anonymization, standardization, and harmonization to ensure their interoperability and future reuse.

In the initial proposal, the smart repository’s architecture involved a centralized solution hosted by the organization responsible for the data lake, but this approach raises compliance concerns regarding the sensitive health data collected in living labs. Since these use cases involve medical applications, the data collected represents health-related information and is therefore classified as personal data under the GDPR.

In the healthcare domain, data ownership and processing responsibilities lie primarily with the healthcare institutions involved. These organizations determine the purposes and means of data processing and are supported by data controllers and, where applicable, Data Protection Officers (DPOs). Because each living lab of DHEAL-COM is operated independently by different institutions, each organization retains full ownership and responsibility for the collected health data. This makes it legally and operationally impractical to consolidate all health data into a centralized repository. However, if the data have been truly anonymized so that reidentification is no longer possible, they are no longer subject to constraints of the GDPR. On the other hand, pseudonymized data are still within the scope of the regulation because they can be reassociated with individuals through auxiliary information, as defined in Article 4(5) of the GDPR. For this reason, two distinct situations must be distinguished when talking about data collected during the project:

Data deriving from the interaction with the patient during the project trials, during the use cases and living labs of the project, for which anonymization is not possible because DHEAL-COM is a territorial project that follows patients over time. If anonymized, these patient-related data would lose their clinical utility and could no longer support the continuity of care;

- Aggregated and/or anonymized data starting from experimental data obtained from patients.
- In the first case, it is not possible to proceed with centralized management by the smart repository, but they must remain available and managed by the organization that owns them. In the second case, the smart repository can transfer and manage them centrally.

As a result, the smart repository is implemented as a distributed system, with each partner organization managing its own local node for acquisition, processing, and storage of health data. The centralized repository, located at the Work Package (WP) leader’s infrastructure, only manages non-personal project data and a collection of metadata used to facilitate federated queries of datasets hosted at local nodes.

**FIGURE 3:**
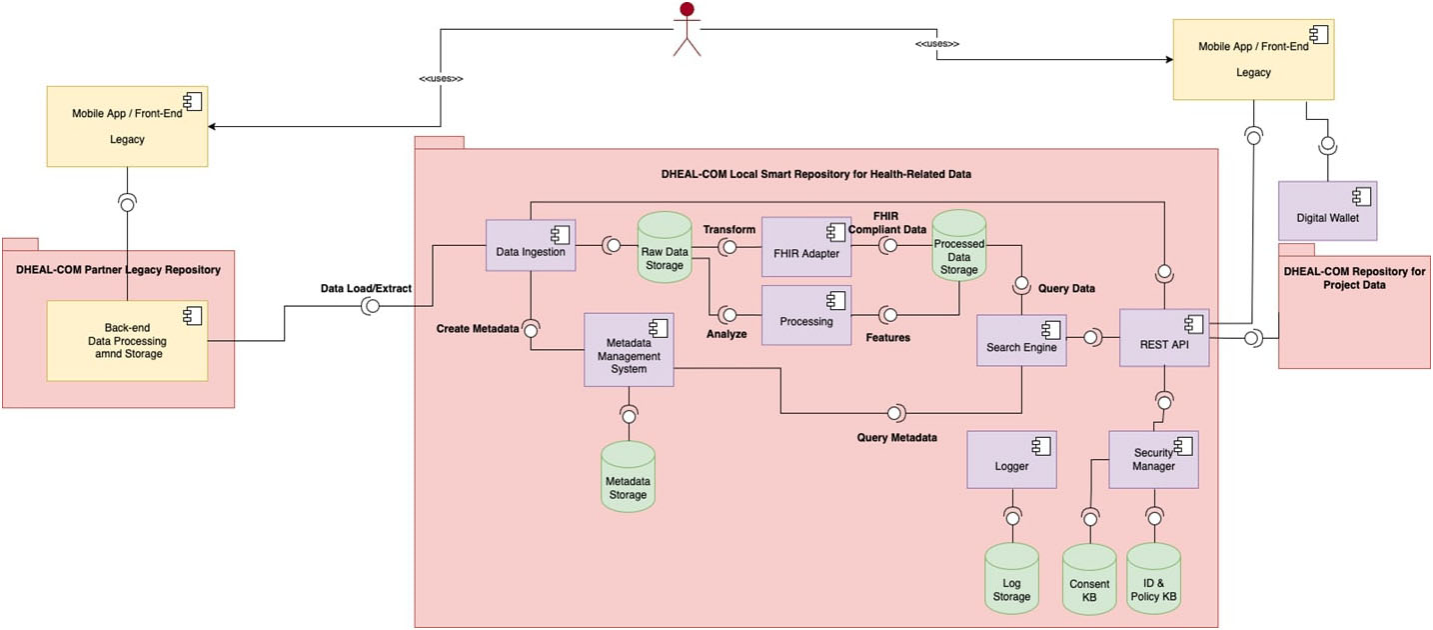
It depicts the architecture of the local nodes. In particular, each partner healthcare organization deploys a local repository and is responsible for managing health-related patient-level data collected during projects trials and living labs.

**FIGURE 4:**
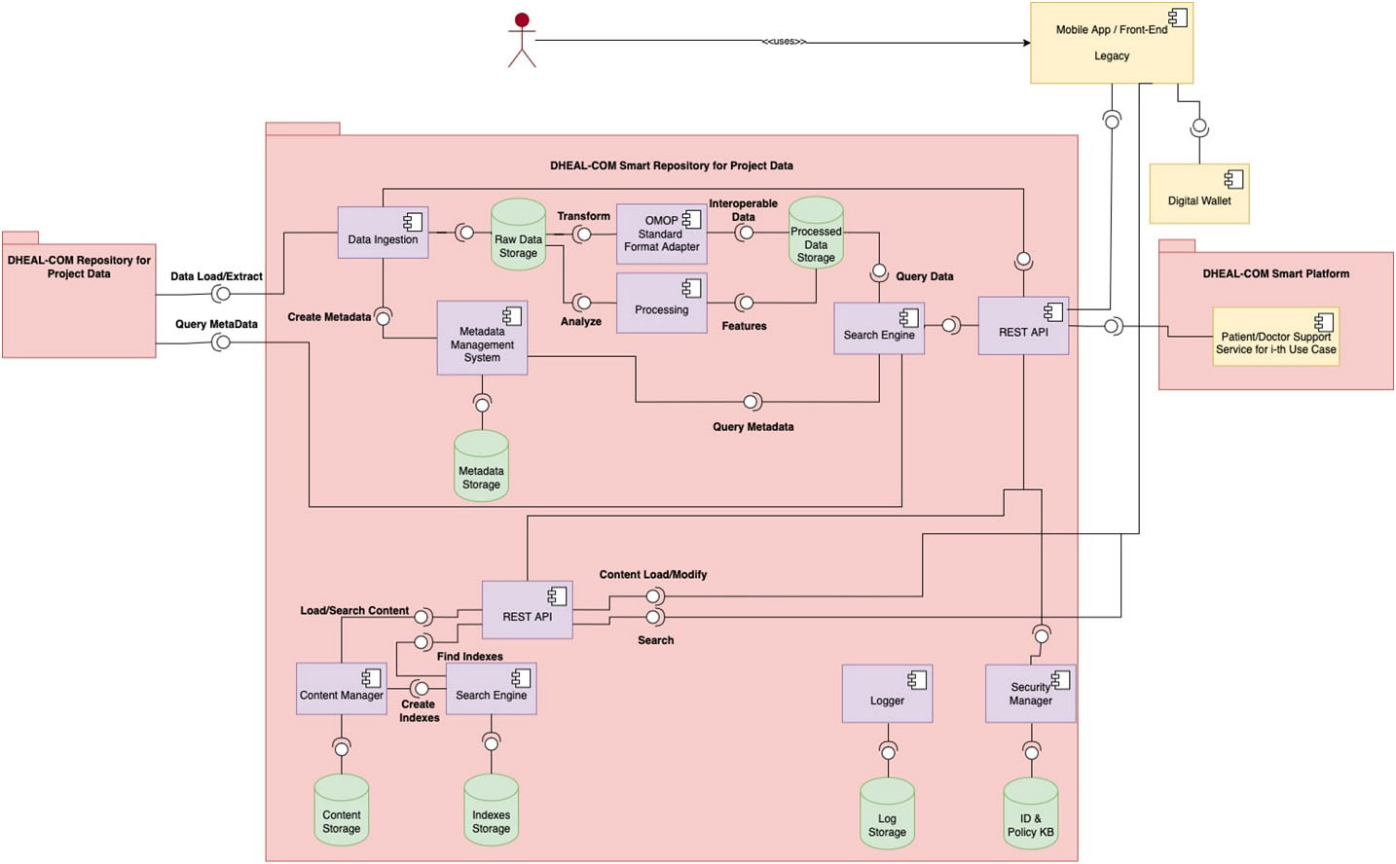
It depicts the architecture of the central repository, which is hosted by the project leader’s infrastructure and manages non-personal project-level data, aggregated/anonymized and synthetic data, and metadata from local nodes.

Figure 1 illustrates the high-level architecture of the Data Lake, showing the interaction between distributed and centralized elements. Figure 3 shows the detailed design of the system architecture for the local nodes running at each partner organization, while Figure 4 depicts the centralized project-level components. To better describe the architectural composition and responsibilities of both the local nodes and the central repository, Table 5 illustrates their respective components and functions. Specifically, the HL7 conversion is done at the local nodes, while the OMOP one at the central node, as it worked on data that are no longer within the GDPR restrictions as anonymized aggregated.

**TABLE 5:** Comparison of technical components of local nodes with respect to the central repository within the federated architecture.

Component	Function	
	Local node	Central repository
Data ingestion	• Interacts with the legacy solution to obtain structured or unstructured health data *ex ante* to the project. • Interacts with the REST API to obtain health data from users either directly or indirectly. • Verifies the correctness and completeness of the data provided, the lack of duplications, that the data source is present in a secure manner. • Populates a KB of raw data, also managing the versioning of modified or updated data. • Interacts with a metadata generation component.	• Interacts with the local solution to obtain structured or unstructured observational or aggregated data, and with users by interacting with the REST API to obtain project data from users or directly. • Verifies the correctness and completeness of the data provided, the lack of duplications, that the data source is present in a secure manner • Populates a KB of raw data, also managing the versioning of modified or updated data. • Interacts with a metadata generation component or provides it with metadata from local repositories.
Raw data storage	It receives data from Data Ingestion and stores it without any processing.	It receives data from Data Ingestion and stores it without any processing.
Metadata management system	It implements solutions to generate metadata and passes it to a dedicated storage solution.	It implements solutions to generate metadata and passes it to a dedicated storage solution.
Format converter	An ETL process converts the data into a standard format, namely FHIR and the IGs implemented starting from HL7 standards. This responds to the interoperability requirement, which stores it in a processed data storage solution.	An ETL process converts the data into a standard format, namely OMOP, and meets the interoperability requirement, which stores them in a processed data storage solution.
Analytics engine	It aggregates the data or determines specific statistical indexes, based on the logic and needs of the organization’s use cases.	It aggregates the data or determines specific statistical indexes, based on the logic and needs of the project use cases.
Search engine	It implements indexing and searching of health data and their metadata.	It implements the indexing and search of healthcare data in a federated manner or of project data and their metadata.
REST API	It supports interaction with the user but also allows interaction with the logically centralized smart repository to provide observational/aggregated data not subject to the GPDR.	It supports user interaction but also allows interaction with the open platform to provide data to applications.
Two cross-platform services: logging and security modules	• The logging component manages and stores logs of the operations performed. • The security component supports the implementation of authentication and authorization solutions for users accessing via the REST API, but also acquires and integrate the consent of the interested parties in the authorization mechanism. This component also has the role of overseeing the persistence of data while the lawfulness of processing continues, and of deleting data when they exhaust the purpose for which the processing was authorized.	• The logging component manages and stores logs of the operations performed. • The security component supports the implementation of authentication and authorization solutions for users accessing via the REST API.

A key aspect of our architecture is data research and retrieval. Local nodes expose a RESTful API for queries, but only within their organization and for accredited users. The centralized node, instead, performs federated queries across local nodes without ever accessing raw patient-level data. In DHEAL-COM, sites will periodically send de-identified batch extracts to a central OMOP hub for analysis. The central node relies on metadata (schema profiles, terminology coverage, data freshness, row counts, privacy settings) to plan and coordinate queries, while all record-level data remain behind local firewall. Metadata itself can pose re-identification risks, and its compliance is subject to risk assessments and DPIAs to ensure safety. For instance, a combination of elements such as exact age, timestamp of data collection, and a rare LOINC code, could allow an external person to infer the identity of a data subject, even without direct identifiers. For this reason, the federated query system incorporates specific safeguards to minimize such risks, applied in practice through the seven-step workflow described in Table 6. Summarizing, the centralized node only searches metadata (capabilities, quality, and policy), plans the query (contacting local nodes or accessing its own data storage), and aggregates non-identifying outputs. All access to record-level data stays local, with disclosure controls being enforced before anything leaves each contacted local organization’s site.

**TABLE 6:** Seven-step workflow for the federated query system, outlining the process for submitting, executing, and aggregating queries while ensuring privacy and data security at each stage..

Step	Description
1. Query submission and validation	A user or service submits a cohort or summary request (e.g., “Adults with T2D on metformin in the last 12 months, return counts by sex and age bands”). The centralized node validates syntax, requester identity/role, data use permissions, and the intended output type (aggregate only vs. site-internal patient lists).
2. Query planning and site capability assessment	The query component of the centralized node consults a registry of site capabilities and metadata: which FHIR resources and terminologies each site supports (e.g., Condition/LOINC/SNOMED), OMOP version, last ETL date, expected cell-size thresholds, and data lag. It chooses eligible nodes and a plan, so that the logical request is transformed into the executable form each node can run. As a central OMOP hub exists, it executes its own copy of the same translated query on its de-identified, hub-resident OMOP—never reaching back to raw site EHRs.
3. Secure distribution of query packages	The component pushes the minimum necessary query package to each site over a mutually authenticated channel (mTLS/VPN). Each request carries short-lived, signed tokens (scoped to aggregate outputs) and a per-site privacy policy manifest (e.g., minimum cell size = 10; DP noise on small strata). No patient-level identifiers are sent to or requested.
4. Local execution on processed data	Inside the site boundary, the local node runs the translated query only against processed data. Joins, filters, and concept set resolution happen locally.
5. Privacy enforcement and aggregate output generation	Before producing outputs, each node enforces its configured privacy rules: any cell below threshold is suppressed or combined, and calibrated differentially private noise for small strata. Each contacted local node returns only non-identifying aggregates (counts, rates, summary stats). If a site cannot produce a compliant result (e.g., all cells suppressed), it returns a policy-compliant null/partial response.
6. Cross-site aggregation and result compilation	The centralized node collects site-level aggregates, performs consistency checks (e.g., schema alignment, unit normalization), aggregates across nodes (sum/weighted stats), and produces the final cross-site result set (tables/figures). Such a result will be also archived so as to be able to respond to future similar queries.
7. Comprehensive logging and audit trial	Every action done to reply to the received query is captured and logged: who requested what, when, which nodes were selected and why, the exact query variants sent, site policy versions, suppression/noise parameters, and hashes of returned aggregates. Logs are immutable and tamper-evident, and no PHI/PII or raw rows are stored in central logs.

In parallel, and on the advice of a bioethics expert, it has been decided that synthetic data will be included in the central repository. As described in Section 5.2, they are not subject to GDPR, since they don’t belong to any human beings. In a later phase of the project, synthetic data will be generated based on real records from the Ligurian HIV Network (53) and will feed the central repository.

To manage the large volume and complexity of health data, both local and central components are deployed using a containerized, microservices architecture based on Docker and Kubernetes. Table 7 outlines the technical and operational benefits of this architectural choice. The adoption of a microservices architecture in the implementation of the DHEAL-COM project solution allows for the creation of modular, scalable, robust and easily evolvable healthcare systems, capable of supporting regulatory requirements, data growth, operational complexity and federated integration between multiple entities over time. This is a strategic choice to ensure continuity, quality, and security in a constantly changing digital ecosystem.

**TABLE 7:** Main advantages of adopting a containerized microservices architecture, such as the one used in the DHEAL-COM project, to support federated health data infrastructure.

Benefit/features	Description
Backup and disaster recovery	Containerization allows the declarative definition of infrastructures and persistent volumes, facilitating the automatic implementation of data snapshots and mounted volumes via persistent storage classes, periodic backups orchestrated via dedicated operators or controllers, and disaster recovery replicable in secondary environments with Infrastructure as Code.
High availability with redundant systems and automatic failover	Thanks to orchestrators such as Kubernetes, it is possible to run container replicas (pods) on multiple nodes, ensuring continuity even in the event of hardware or software failure, automate the restart of services in the event of an error (self-healing), and implement load balancing and transparent failover for distributed services and databases.
Continuous monitoring with alerts and diagnostics	Containers are natively observable through integrated tools such as Prometheus, Grafana, ELK/EFK that allow the tracking of logs, metrics and alerts. There is also the possibility of integrating Health Check and Readiness Probe to monitor the real status of applications and solutions are available for the support of distributed tracing (e.g., Jaeger) for complex diagnostic analyses.
Fast response times even on large data volumes	Containerized architectures allow you to horizontally scale computational components (API, engine, DB) to manage high-intensity queries, use data caching and distributed microservices optimized for performance, and reduce startup time thanks to the lightness and modularity of containers.
Management of load peaks	With containerization, you can manage variable loads thanks to dynamic autoscaling of pods based on CPU, memory or custom metrics, elastic allocation of resources on available nodes, and deployment in hybrid environments (on-prem/cloud) to temporarily absorb high peaks.
Separation of responsibilities and modularity	Each microservice is designed to manage a specific function (e.g., patient management, prescriptions, reports, informed consent), allowing clarity of responsibility between teams and modules, ease of updating, testing and maintenance, and independent evolution of individual components without impacting the entire system.
Granular horizontal scalability	The microservices architecture allows selectively scaling only the components subject to high loads, optimizing the use of infrastructure resources, and dynamically adapting to load peaks in targeted functional areas (e.g., simultaneous access to the document repository or authentication services).
Resilience and fault isolation	In a distributed system, a failure in a microservice does not compromise the entire system given the possibility of implementing circuit breakers, retry and fallback, fault isolation at the level of individual services or nodes, and improved operational continuity even in the presence of partial anomalies.
Interoperability and heterogeneous integration	Each microservice can expose RESTful, FHIR or gRPC APIs interoperable with third-party systems, be written in different languages and technologies, as long as it communicates via standard protocols, and facilitate integration with legacy systems or federated systems.
Time-to-market and continuous delivery	The microservices model allows for frequent and independent deployment of individual components, ease of applying DevOps and CI/CD pipelines, and reduction of release times thanks to the lower interdependency between modules.
Distributed security and governance	Each service can be protected through fine-grained authentication/authorization (OAuth2, JWT, RBAC), dedicated security policies for sensitive data, and perimeter monitoring and tracking of inter-service traffic (e.g., via service mesh).
Ease of maintenance and technological evolution	Ability to update or rewrite individual services without re-engineering the entire system and progressive adoption of new technologies, frameworks or databases in targeted contexts.

Benefits include high availability, scalability, and enhanced security. Each microservice is independently deployable and maintainable, enabling rapid development cycles, interoperability with heterogeneous systems, and robustness in high-demand or failure scenarios.

## 6 Discussion

The establishment of the EHDS represents a transformative moment for all EU MS. The introduction of a new European digital infrastructure can be seen as a push that forces nations to undertake reforms and actions that, until now, had been postponed due to structural, financial, or political constraints. Achieving EHDS will require investment in national-level digitalization and European interoperable infrastructure. The EU MS themselves and the Commission will support these efforts with dedicated funds, including 12 billion euros from the Recovery and Resilience Facility (RRF) and more than 800 million euros from European programs such as EU4Health, Digital Europe Programme and Horizon Europe (54). In addition, it is reasonable to assume that the EU will support the EU MS not only with funding, but also through clear guidelines, needed to adapt local HIS to shared frameworks.

This context reinforces the necessity for effective synergy between the European and national levels. EHDS cannot succeed unless individual countries can translate European principles into concrete strategies. At the same time, what happens in the national territories will have to help shape European standards and evolution. In this sense, the Italian case assumes particular relevance. The complex organization of the NHS, based on a federated model with strong regional autonomies, makes Italy a paradigm for challenges that other EU MS may face. The Italian healthcare landscape, composed of 20 regional services and further complicated by the ongoing *Autonomia differenziata*, shows all the typical challenges of fragmented information, uneven technological maturity, and weak central governance. There is a concrete risk that this new wave of decentralization will exacerbate regional disparities, further hindering the consistent adoption of interoperable architectures across the country.

Within this fragmented context, the Italian FSE represents an important tool in the evolution of Italy’s digital health ecosystem. Each of the 20 Italian regions has developed its own version of the FSE, reflecting local governance models, technical infrastructures, and implementation timelines. To manage the 20 separated FSE, a core element is the INI, which functions as a document-based infrastructure designed to manage the flow of exchanged documents, even at the interregional level. While this approach addresses the technical challenge of facilitating document exchange, it does not fully support semantic interoperability. Many documents remain unstructured or lack standardized coding, making automatic interpretation or reuse of clinical information difficult. Therefore, INI is an important step toward national technical interoperability, but it is not sufficient to support advanced services such as data analytics or the secondary use of data for research and public health planning. This gap is where the new infrastructure proposed for the EDS comes into play.

In this scenario, semantic interoperability becomes a fundamental requirement. Although Italy has adopted international standards such as HL7 CDA and is gradually migrating to HL7 FHIR, there remains a deep gap in the ability of regions to embrace these standards. The problem is not only syntactic, but also semantic: many regions continue to use local vocabularies, proprietary encodings and data models that are not aligned with national or international standards. The terminology issue thus becomes central. Italy, to date, has not purchased a national license for SNOMED CT, limiting the use of one of the main tools for ensuring semantic interoperability. However, initiatives such as the International Patient Summary (IPS), developed by HL7 International, allow even unlicensed countries to use predefined subsets of SNOMED codes to facilitate minimal data sharing across borders. This approach represents a first step toward shared European terminology services. Semantic interoperability can be supported through standardized terminology services (55), which enable local terminologies to be mapped to national and international coding systems. Initiatives such as ELGA (56), Austria’s EHR system, demonstrate how centralization of terminology services and mapping from CDA to FHIR can be essential tools to facilitate the transition to an interoperable ecosystem (57).

In Italy, an important part of the standards adopted has historically been driven by administrative needs. In the hospital discharge process, two distinct documents are generated: the hospital discharge report, which is the document given to the patient upon discharge from the hospital, and the *Scheda di Dimissione Ospedaliera* (SDO), which is the official discharge report sent to the Ministry. The case of the SDO is emblematic: its standard format is widely used because it is required for reimbursement by the Ministry. The economic factor has encouraged the use of solutions focused more on administrative accounting than on patient care. The hope is that EHDS can contribute to a shift in perspective, switching the focus from administration to the reuse of clinical data for health, research and public health purposes. It is precisely the reuse of data that represents one of the most innovative and promising elements of the EHDS. The ability to enhance the value of secondary clinical data may act as a lever to incentivize standardization. In this context, integration between exchange standards, such as HL7, and data models for reuse, such as OMOP, will become increasingly crucial. The future architecture of health information systems will need to be able to support both levels: on the one hand, interoperable communication between clinical systems; on the other hand, standardized analysis for research and governance.

The most recent Italian initiatives, such as the DHEAL-COM project, show the potential for synergy among academia, industry, and public administration. Such projects not only promote the experimentation of new models but also create the basis for developing new technical specifications to be proposed to HL7 Italy working tables (58), actively contributing to the definition of national IGs. These collaborative efforts help align local experiences with broader European goals. In this context, DHEAL-COM and the EHDS framework are evolving in parallel. Since the project began while the EHDS regulation was still taking shape, some aspects, such as roles, responsibilities, and access bodies, remain to be defined. To stay aligned, DHEAL-COM has adopted a flexible approach to satisfy future compliance. As the regulatory framework becomes clearer, the project will progressively define and share a compliance matrix and reusable technical resources.

However, none of these technical advances can succeed without addressing data protection and privacy concerns. In the Italian context, the *Garante* plays a pivotal role. The *Garante* adopts a conservative stance, particularly regarding secondary data use, cross-border data sharing, and anonymization practices. This cautious approach, while essential for protecting fundamental rights, can also introduce delays and additional costs, particularly for public and research initiatives involving large-scale health data. A key point is that the *Garante*’s decisions and guidelines, while formally not equivalent to legislations, have a regulatory effect in practice. This reflects a broader governance issue: although the GDPR sets a harmonized legal framework, each national DPA retains wide discretion to issue guidelines, authorizations and biding decisions. For organizations engaged in pan-European projects, the challenge lies in navigating these evolving and sometimes divergent national interpretations, which increase compliance burdens and risk hindering the development of an integrated European health data infrastructure.

Moreover, no transformation will be possible without a significant investment in skills. Interoperability and standardization are not just about developers and engineers, but also about clinicians, researchers, public decision makers, healthcare professionals, and final users. What is needed is widespread, multilevel education that enables all actors in the system to understand the existence and value of standards. This is one effective way to foster a data culture that supports informed decision making and encourages the adoption of interoperable, patient-centered solutions.

Italy’s journey toward EHDS compliance is both a national challenge and a European opportunity. True progress will require not only technical solutions, but also strong governance, legal clarity, and engagement of different stakeholders. Only through this collective effort will it be possible to build a truly interoperable and resilient EHDS.

## Data Availability

The original contributions presented in the study are included in the article/supplementary material, further inquiries can be directed to the corresponding author.
